# Gardening, healthy aging, and longevity: Longitudinal evidence from 25 years of the Lothian Birth Cohort 1921

**DOI:** 10.1016/j.jenvp.2025.102889

**Published:** 2025-12-23

**Authors:** Janie Corley, Alison Pattie, Sarah E. Harris, Ian J. Deary, Simon R. Cox

**Affiliations:** a Lothian Birth Cohorts, Department of Psychology, University of Edinburgh, Scotland, UK; b Edinburgh Futures Institute, University of Edinburgh, Scotland, UK

**Keywords:** Gardening, Healthy aging, Longitudinal birth cohort, Gait speed, Telomere length, Mortality

## Abstract

Gardening is a common leisure activity among older adults, yet its potential to support healthy aging remains underexplored, particularly in longitudinal contexts and across multiple aging domains. This study investigated whether gardening frequency was associated with psychological, physical, and biological aging markers, as well as mortality, in the Lothian Birth Cohort 1921 (LBC1921). Gardening frequency was assessed via questionnaire at a mean age of 79 years (baseline: 1999–2001) in 475 participants. Aging markers were measured at 79, 83, 87, and 90 years. Psychological markers (quality of life, psychological wellbeing) were self-reported; physical function markers (lung function, gait speed, grip strength, functional ability) were objectively assessed; and biological markers (telomere length, DNA methylation-based PhenoAge) were blood-derived. Survival was tracked using 25-year mortality linkage data. Analyses included linear regression, growth curve modeling, and Cox proportional hazards, adjusted for individual- and neighbourhood-level covariates. Of the sample, 207 gardened frequently, 78 sometimes, and 190 never or rarely. At baseline (age 79), higher gardening frequency was associated with better psychological wellbeing, stronger physical function, and longer telomeres. Longitudinally, more frequent gardening predicted slower declines in gait speed and telomere attrition from age 79 to 90. Frequent gardeners had a 22 % lower mortality risk (HR = 0.78, 95 % CI: 0.62–0.97) than those who never or rarely gardened. Associations were not substantially confounded by sociodemographic, lifestyle, or physical activity factors. Our results suggest that gardening may support wellbeing and longevity, with potential implications for aging in place for older adults.

## 1. Introduction

With the global population of older adults rising rapidly, supporting healthy aging has become an urgent public health priority (Gianfredi et al., 2025). By 2030, one in six people worldwide will be aged 60 or over, and this number is expected to double to 2.1 billion by 2050 (World Health Organization, 2022). In the UK, nearly one in five people are aged 65 and over, with many experiencing multiple chronic health conditions, functional decline and cognitive impairment (Centre for Ageing Better, 2024). These demographic trends highlight the growing need for preventive, low-cost, and scalable lifestyle interventions to promote health and independence in later life. Gardening may represent one such intervention, with growing evidence suggesting it supports health and wellbeing across the lifespan, including benefits to physical health, positive affect, life satisfaction, and increased engagement in physical and social activities (Chalmin-Pui et al., 2021; Cheng et al., 2010; Cheng & Pegg, 2016; Fjaestad et al., 2023; Genter et al., 2015; Gulyas et al., 2024; Harding et al., 2022; Hassan et al., 2018; Howarth et al., 2020; Kingsley et al., 2023; Krols et al., 2022; Kunpeuk et al., 2020; Panțiru et al., 2024; Scott et al., 2015; Scott et al., 2020; Shiue, 2016; Soga et al., 2017; Wang et al., 2024; Wang & Boros, 2025; Wang & MacMillan, 2013). These benefits may in turn support healthier aging and help mitigate age-related declines, which is the focus of the current study.

As an activity typically carried out around or near the home, gardening may also promote autonomy and a sense of connection to the local environment, both key components of ‘aging in place’ (Pani-Harreman et al., 2021). Additionally, gardening provides direct exposure to green space, which is independently linked to mental wellbeing, lower mortality risk, and reduced incidence of chronic disease in later life (De Keijzer et al., 2020; Twohig-Bennett & Jones, 2018), suggesting that gardening may deliver benefits through both behavioural and environmental pathways.

Despite the promising findings to date, systematic reviews and meta-analyses highlight several limitations in the current literature (Howarth et al., 2020; Panțiru et al., 2024; Soga et al., 2017; Wang & MacMillan, 2013; Sia et al., 2020; Van den Berg & Custers, 2011). Many studies rely on cross-sectional designs, self-reported outcomes, or small and unrepresentative samples, limiting conclusions about causality or long-term effects. Objective indicators—including directly measured physical function and blood-based biomarkers—are rarely included, and longitudinal research remains scarce. Moreover, while gardening is a particularly popular leisure activity among older adults, few quantitative studies have specifically addressed this demographic (Wang & MacMillan, 2013). Existing studies in older samples tend to focus on general health or psychosocial outcomes, leaving other important domains of aging, such as cognitive function or biological aging, largely unexplored (Machida, 2019; Scott et al., 2020; Sommerfeld et al., 2010; Van den Berg et al., 2010). For example, no studies to date have examined gardening in relation to telomere length, a key biomarker of cellular aging (Chakravarti et al., 2021; Eppard et al., 2024; Rossiello et al., 2022).

Horticultural programmes in residential care settings further support the health-related benefits of gardening in later life. Studies across North America, the UK, and Japan have reported pre-post improvements in depression, anxiety, social relationships, and physical fitness (e.g., aerobic endurance, agility) following gardening activity sessions (Nicholas et al., 2019; Park, Lee, Lee, et al., 2016; Wang et al., 2022). However, this evidence comes mainly from small, short-term studies in clinical or institutional populations, limiting generalisability to community-dwelling older adults (Yun et al., 2024).

A further challenge in evaluating the potential benefits of gardening is the multifactorial nature of health and aging, which are influenced by a wide range of interrelated social, environmental, and behavioural factors (Castruita et al., 2022). Variables such as education, socioeconomic status, neighbourhood quality, gender, and health behaviours (e.g., smoking, physical activity, and body mass index) are known correlates of health outcomes (Hemadeh et al., 2025; Mutz et al., 2021; Ng et al., 2020; Yoshida et al., 2022). If gardening behaviours are causally related to healthier aging (rather than simply being another indicator of general good health) then such associations would be expected to remain robust after adjustment for these covariates, which themselves offer quantitative indicators of health. Such correlational data are in a position to disprove, though not in a position to prove, causality. This makes the availability of suitable datasets particularly important. Large, prospective, community-based longitudinal studies of older adults that collect comprehensive life-course data provide valuable opportunities to test these relationships and characterise change in health outcomes over time. Understanding whether and how gardening supports healthy aging has implications for researchers, clinicians, and policymakers seeking accessible and sustainable interventions to promote wellbeing in older age.

Recent prospective studies of aging increasingly highlight the role of modifiable lifestyle behaviours in supporting mental and physical health in later life (Lafortune et al., 2016; Peters et al., 2019; Thomas et al., 2023). Building on this, our previous work with the same well-characterised cohort used in the current study, showed that gardening was associated with more favourable cognitive aging (Corley et al., 2024), indicating potential benefits that extend beyond commonly studied outcomes such as emotional health.

In the present study, we investigate whether gardening frequency is associated with baseline levels and trajectories of a broader set of aging markers—psychological wellbeing, physical function, biological aging, and mortality risk. We use data from the Lothian Birth Cohort 1921 (LBC1921)—a rich, longitudinal study following individuals from early old age into their 90s—with repeated health assessments over more than a decade, allowing examination of changes in key aging markers, alongside verified mortality linkage data. Baseline data on gardening activity, physical activity, and contextual factors such as socioeconomic background, lifestyle, and neighbourhood quality, enable adjustment for important potential confounders. Drawing on one of the longest-running aging cohorts worldwide, this study offers unique insight into how everyday interactions with the environment may contribute both to day-to-day wellbeing and to long-term survival.

## 2. Methods

### 2.1. Lothian Birth Cohort 1921 (LBC1921)

Participants were drawn from the Lothian Birth Cohort 1921 (LBC1921), a longitudinal study of aging that began in 1999–2001, when members were approximately 79 years old (Wave 1; baseline for the present analyses). Follow-up assessments occurred at mean ages 83 (Wave 2), 87 (Wave 3), and 90 (Wave 4). The cohort comprises 550 individuals (234 men and 316 women), most of whom had participated in the Scottish Mental Survey of 1932, which tested nearly all 11-year-olds in Scotland (Scottish Council for Research in Education, 1933). At baseline and follow-up waves, participants completed cognitive, psychological, and medical assessments, as well as questionnaires on personality, wellbeing, and health behaviours (Deary et al., 2009, 2012; Taylor et al., 2018). For the present analyses, participants with available gardening data at baseline (age 79) were included. After excluding 75 individuals with missing gardening data, the analytic sample comprised 475 participants.

Ethical approval for the LBC1921 study was provided by the Lothian Research Ethics Committee for test Waves 1–3 at ages 79, 83 and 87 (LREC/1998/4/183, LREC/2003/7/23, 1702/98/4/183) and by the Scotland A Research Ethics Committee for test Wave 4 at age 90 (10/MRE00/87, 10/MRE00/87). All participants provided written informed consent.

### 2.2. Assessment of gardening

At baseline (age 79), participants indicated how often they engaged in gardening (“never,” “rarely,” “sometimes,” “frequently”) as part of a lifestyle questionnaire, previously described by Gow et al. (2017). Due to few responses in the “rarely” category, “never” and “rarely” were combined to form a three-level variable: 0 = never/rarely, 1 = sometimes, 2 = frequently, with higher values indicating greater gardening frequency.

### 2.3. Assessment of Psychological Aging

#### Quality of Life (QoL):

Measured at ages 79 and 90 using the WHOQOL-BREF (WHOQOL Group, 1998), a 26-item measure covering physical, psychological, social, and environmental domains. Items were rated on a 5-point scale and transformed to a 0–20 scale following the WHO manual (WHO, 1997). Domain scores were summed to create an overall QoL score, with higher values reflecting better QoL.

#### Psychological Wellbeing:

Measured at ages 79, 87, and 90 using the 14-item Hospital Anxiety and Depression Scale (HADS; Zigmond & Snaith, 1983). Items were scored on a 4-point scale (0–3 per item), with a maximum total score of 42. Scores were reverse-coded so that higher values indicated better self-reported mental health.

### 2.4. Assessment of Physical Aging

Trained nurses assessed lung function, gait speed, grip strength, and functional ability at ages 79, 87, and 90; grip strength was additionally measured at age 83. These objective measures are established indicators of healthy aging (Lara et al., 2015; Wagner et al., 2016).

#### Lung Function:

Forced expiratory volume from the lungs in 1 s (FEV_1_) was measured using a Micro Medical Spirometer (best of three attempts). FEV_1_ indicates respiratory health in older adults and is independently associated with activities of daily living, hospitalisation, and mortality risk (Hegendörfer et al., 2017).

#### Gait Speed:

Time to walk 6 m (in seconds) at a normal pace was recorded. Slower gait speed indicates poorer mobility and higher mortality risk (Kim et al., 2016; Studenski et al., 2011). Scores were reverse-coded so that higher values indicate better walking speed.

#### Grip Strength:

Measured using a Jamar Hydraulic Hand Dynamometer (best of three attempts, dominant hand). Grip strength is an indicator of muscular strength and overall physical function and predicts future health outcomes amongst older adults (Soysal et al., 2021).

#### Functional Ability:

Measured using the 9–item Townsend Scale (Townsend, 1979) assessing difficulties with basic and instrumental activities of daily living (ADLs) such as dressing, bathing, preparing meals, shopping, and managing finances. Items were scored from 0 (no difficulty) to 2 (unable to complete), with a maximum score of 18, reverse-coded so higher values indicate better functional ability.

### 2.5. Assessment of biological aging

#### Telomere Length:

Measured from peripheral blood DNA using quantitative real-time polymerase chain reaction (qPCR) following Martin-Ruiz et al. (2004). Four internal control samples with known telomere lengths were included on each plate to correct for variation and allow calculation of absolute telomere lengths from relative telomere-to-single-copy gene (T/S) ratios. Samples were assayed in quadruplicate, and mean values were used for analysis.

#### DNA Methylation-based PhenoAge (DNAm PhenoAge):

An epigenetic biomarker of biological aging was calculated using Levine et al.’s algorithm (Levine et al., 2018) implemented in Horvath’s online calculator. Scores were reverse-coded so that higher values reflect healthier biological aging (i.e., slower age acceleration).

### 2.6. Assessment of mortality

Mortality follow-up was based on ongoing linkage to national death records and extended from baseline (1999) to the current analyses (2025), including all eligible participants with baseline gardening data (*n* = 475). As registry-based mortality data are continuously updated and do not depend on in-person attendance, all cohort members contributed to the mortality analyses regardless of participation in later waves. Of the 475 participants, 473 had valid linkage data; 469 were deceased, and 4 were alive (aged 103). Two participants were unable to be linked due to relocation outside Scotland, and excluded from the survival analysis. Survival time was calculated as age at death or censoring (in days).

### 2.7. Covariates

Analyses adjusted for baseline age (days), sex, education (years), occupational social class, living alone (yes/no), body mass index (BMI), smoking status (ever/never), self-reported history of cardiovascular disease, stroke, or cancer (yes/no), and neighbourhood quality. Occupational social class was based on participants’ highest-status occupation, classified according to the 1951 Classification of Occupations (General Register Office, 1956: I professional; II managerial/technical; III skilled; IV semi-skilled; V unskilled). For women, husband’s occupation was used if it indicated a higher social class. Neighbourhood quality reflected perceived accessibility to local amenities (e.g., GP, post office, parks, grocery stores, sports facilities) within walking distance (defined as approximately half a mile from the home), based on questionnaire items from the Scottish Household Survey 1999; Scottish Executive, 2000). Height (cm) was additionally included in physical function (lung function, gait speed, grip strength) models. Baseline physical activity (days per month exercising >20 min) was included in sensitivity analyses to examine whether any associations between gardening and healthy aging were explained by higher overall activity levels.

### 2.8. Statistical analysis

Analyses were performed in R 4.3.3 (R Core Team, 2024) using ‘lavaan’ for growth curve modeling (Rosseel, 2012), ‘lm’ for regression, and ‘survival’ for Cox proportional hazards models. Continuous variables were standardised and gardening frequency was modeled as a three–level variable (never/rarely, sometimes, frequently). Outcomes were coded so that higher values indicated better health or slower aging trajectories.

We used latent growth curve models to examine associations between gardening (exposure) and aging markers (outcomes) within a structural equation modeling framework. These models simultaneously estimate intercepts (baseline levels, age 79) and slopes (rates of change, age 79 to 90) in outcome measures across the follow-up period. Missing data were handled using full information maximum likelihood (FIML), which assumes data are missing at random and incorporates all available data points, thereby reducing bias from incomplete observations. Path weights reflected mean time lags from baseline to follow-ups (4.29, 7.54, and 11.06 years). Model fit was assessed using Comparative Fit Index (CFI >0.90 acceptable; >0.95 good), Root Mean Square Error of Approximation (RMSEA <0.06 acceptable), and Standardised Root Mean Square Residual (SRMR <0.08 acceptable) (Hu & Bentler, 1998).

Quality of life (QoL), measured at only two waves (ages 79 and 90), did not meet the minimum number of time points required for latent growth curve modeling. Therefore, we analysed QoL using regression models examining baseline associations, and change scores calculated as residuals from regressing age 90 on age 79 scores.

Mortality was modeled using Cox proportional hazards, with time-to-event defined as age at death or censoring at the date of analysis (in days). The reference group was never/rarely gardening.

Three models were tested: Model 1: age, sex; Model 2: multivariable (age, sex, education, occupational social class, living alone, BMI, smoking, CVD, stroke, cancer, neighbourhood quality); Sensitivity analysis: Model 2 + physical activity. Results are presented as standardised β (beta) coefficients with standard errors, *p*–values, and 95 % confidence intervals (CI) are reported. False Discovery Rate (FDR) correction was applied across all tests (q < 0.05; Benjamini & Hochberg, 1995).

## 3. Results

### 3.1. Sample characteristics

Participants were members of the LBC1921, with baseline data collected at mean age 79 (Wave 1, *n* = 550), which served as the starting point for all analyses. After excluding 75 individuals with missing gardening data, the baseline analytic sample comprised 475 participants (57 % women; mean age = 79.06 years, SD = 0.6). Follow-up assessments occurred at mean ages 83 (*n* = 298), 87 (*n* = 198), and 90 (*n* = 126), with an average follow–up duration of 11.1 years (SD = 0.5). A flowchart illustrating participant inclusion and follow–up at each wave is provided in Supplementary Fig. S1.

Gardening frequency was originally recorded in four categories (never, rarely, sometimes, frequently). Due to low numbers of participants in the “rarely*”* category (*n* = 48, 10.1 %), this was combined with “never” (*n* = 142, 29.8 %) to improve model stability. Model fit indices (e.g., Akaike Information Criterion; AIC) supported the three-level categorisation as a better fit across all adjusted models in the main analyses than the original four-level variable. The final categorisation used in analyses included: never/rarely (*n* = 190, 39.9 %); sometimes (*n* = 78, 16.4 %); and frequently (*n* = 207, 43.6 %).

Table 1 presents sample characteristics by gardening frequency. Participants who reported gardening frequently tended to have higher occupational social class, better perceived neighbourhood quality, and greater physical activity levels. Years of education were higher among those who gardened sometimes or frequently compared with those who never or rarely gardened. BMI was lowest among those who gardening sometimes. Missing covariate data are detailed in Supplementary Table S1. Table 2 shows outcome measure summary statistics at each wave. Most aging markers declined over time (QoL, lung function, gait speed, grip strength, functional ability, telomere length, (i.e., shortened), DNAm PhenoAge (i.e., increased), while psychological wellbeing improved slightly.

**Fig. 1.**
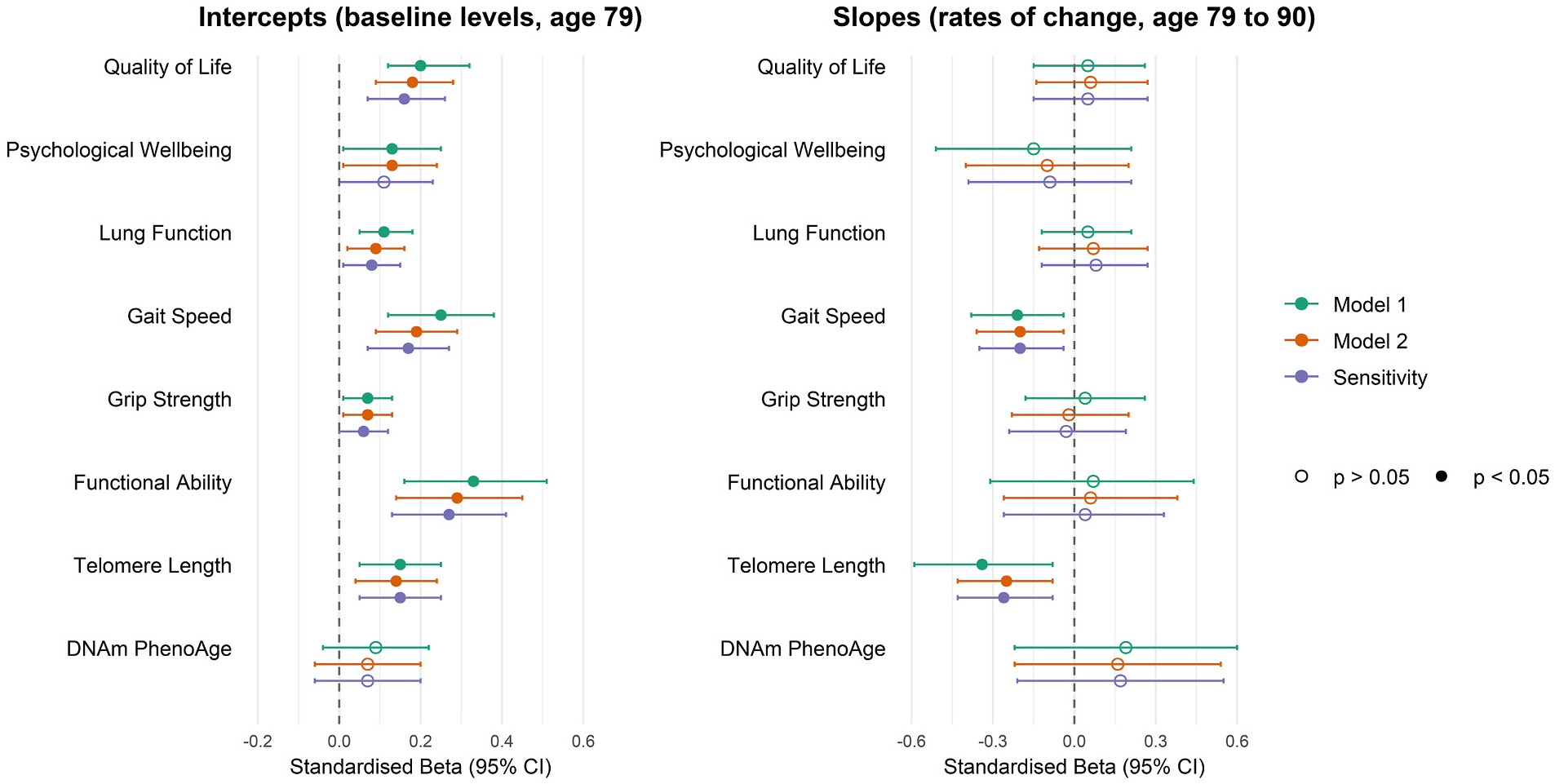
Associations Between Gardening Frequency and Aging Outcomes. Forest plots showing associations between gardening frequency and aging outcomes (corresponding values reported in Table 3). Standardised beta coefficients and 95 % confidence intervals (CI) are displayed for each outcome. Outcomes were coded such that higher values indicate healthier aging. For reverse-coded measures (Psychological Wellbeing, Gait Speed, Functional Ability, DNAm PhenoAge), negative slopes indicate less decline or slower aging associated gardening over time. For Telomere Length, a negative slope reflects slower telomere attrition over time, corresponding to healthier aging. Model 1: adjusted for age and sex; Model 2: additionally adjusted for education, social class, living alone, neighbourhood amenities, smoking, body mass index, cardiovascular disease, stroke, and cancer; Sensitivity: Model 2 plus physical activity. Statistically significant associations (*p* < 0.05) are indicated by filled points.

Table 3 reports associations between gardening frequency and modeled aging outcomes, estimating intercepts (baseline levels) and slopes (rate of change). Results reported in the text refer to the multivariable–adjusted model (Model 2); sensitivity analyses with additional adjustment for physical activity are shown for comparison. Overall model fit was acceptable across all models and outcomes (see Supplementary Table S2). Fig. 1 presents the corresponding forest plots for the main analyses, displaying effect sizes for both intercept and slope associations for each outcome.

### 3.2. Gardening and aging outcomes: intercepts (age 79) and slopes (age 79 to 90)

#### Psychological Aging:

More frequent gardening was associated with higher baseline overall QoL at age 79 (β = 0.177, 95 % CI [0.090, 0.283]), and with the following QoL sub-domains; physical QoL (β = 0.186, 95 % CI [0.102, 0.300]); psychological QoL (β = 0.145, 95 % CI [0.061, 0.263]); and environmental QoL (β = 0.121, 95 % CI [0.035, 0.225]). Social QoL was significant in the age- and sex-adjusted model but not in the multivariable model (β = 0.086, 95 % CI [−0.011, 0.191]). Full results for the QoL sub-domains are presented in Supplementary Table S3. Gardening was also positively associated with greater psychological wellbeing (β = 0.125, 95 % CI [0.010, 0.240]), i.e., fewer symptoms of anxiety or depression.

Gardening was not significantly associated with longitudinal changes in overall QoL from age 79 to 90 (β = 0.058, 95 % CI [−0.144, 0.272]) or its domains (physical QoL: β = −0.001, 95 % CI [−0.203, 0.201]; psychological QoL: β = 0.016, 95 % CI [−0.192, 0.227]; social QoL: β = 0.099, 95 % CI [−0.102, 0.316]; environmental QoL: β = 0.121, 95 % CI [−0.067, 0.339]), nor with change in psychological wellbeing (reverse-coded; β = −0.101, 95 % CI [−0.401, 0.199]).

#### Physical Aging:

Gardening frequency was positively associated with baseline lung function (β = 0.092, 95 % CI [0.023, 0.160]), gait speed (β = 0.192, 95 % CI [0.090, 0.294]), grip strength (β = 0.070, 95 % CI [0.015, 0.130]), and functional ability (β = 0.293, 95 % CI [0.140, 0.446]).

Higher gardening frequency was also associated with a slower decline in gait speed from age 79 to 90 (reverse-coded; β = −0.200, 95 % CI [−0.357,−0.081]). No significant slope associations were observed for lung function (β = 0.072, 95 % CI [−0.128, 0.272]), grip strength (β = −0.015, 95 % CI [−0.227, 0.198]), or functional ability (β = −0.060, 95 % CI [−0.256, 0.376]).

#### Biological Aging:

More frequent gardening was associated with longer baseline telomeres (β = 0.142, 95 % CI [0.045, 0.239]) and slower telomere attrition over time (β = −0.252, 95 % CI [−0.429, −0.075]). Associations with DNAm PhenoAge were in the expected direction but not statistically significant at baseline (β = 0.070, 95 % CI [−0.062, 0.201]) or longitudinally (β = 0.160, 95 % CI [−0.221, 0.542]).

### 3.3. Sensitivity analyses: adjustment for physical activity

Further adjustment for physical activity did not substantially alter associations between gardening and aging outcomes (Table 3, Sensitivity Model). Attenuation was minimal, averaging around 10 % across models. All associations that were significant in Model 2 remained robust, with the exception of psychological wellbeing, for which the effect was reduced and no longer statistically significant (β = 0.114, 95 % CI [−0.001, 0.229]).

### 3.4. Gardening and mortality

**Fig. 2.**
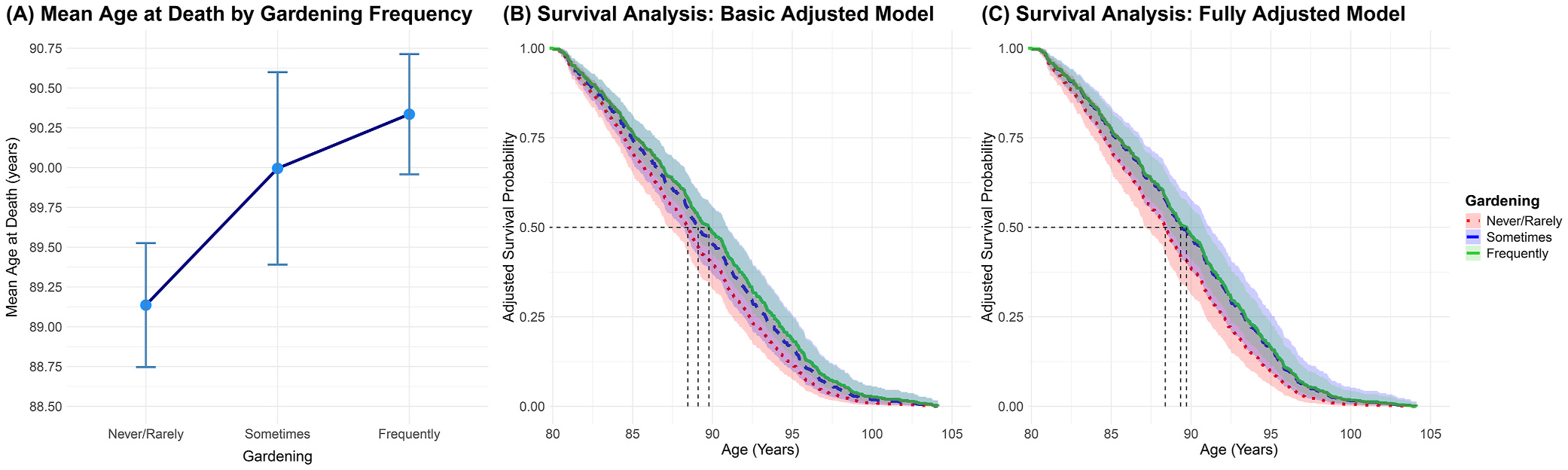
Survival by gardening frequency. Panel A shows mean age at death by gardening frequency; error bars represent standard errors. Panel B shows the age- and sex adjusted Kaplan–Meier survival probability curves by gardening frequency group. Panel C shows the multivariable-adjusted (including physical activity) survival probability curves by gardening frequency group. Shaded areas around each line indicate 95 % confidence intervals. Black dotted lines mark the median survival age (the point at which survival probability = 50 %). Horizontal and vertical reference lines extend from the 50 % survival point to the x-axis (age in years), representing the time at which median survival occurs. Median survival times: Panel A: 88.5, 89.1, 89.8 years; Panel B - 88.4, 89.3, 89.7 years, for never/rarely, sometimes, frequently, respectively.

By the end of follow–up, 469 of 473 participants (99 %) had died and four were still alive. Mean survival age was 89.8 years (SD = 5.4). Survival age increased with gardening frequency: 89.1 years (SD = 5.4) for participants who never or rarely gardened; 90.0 years (SD = 5.3) for those gardening sometimes; and 90.3 years (SD = 5.4) for those gardening frequently (Fig. 2, panel A).

In Cox proportional hazards models (*n* = 473, Table 4), frequent gardening was associated with a significantly lower mortality risk compared to never/rarely gardening (Model 1: HR = 0.78, 95 % CI [0.640, 0.961]; Model 2: HR = 0.77, 95 % CI [0.622, 0.965]). Associations remained significant after further adjustment for physical activity (HR = 0.78, 95 % CI [0.624, 0.972]) indicating that frequent gardeners had a 22 % lower hazard of death, even after accounting for age, sex, education, social class, living alone, neighbourhood amenities, smoking, BMI, CVD, stroke, cancer, and physical activity. Sometimes gardeners had a 20 % lower hazard of death (HR = 0.80, 95 % CI [0.601, 1.054]) in the final model compared with the reference group, but this was not statistically significant (*p* = 0.111). Model fit was acceptable (Likelihood Ratio χ^2^(14) = 42.57, *p* < 0.001); concordance index = 0.60). Kaplan–Meier survival curves (Fig. 2, panels B and C) showed a graded survival advantage with increasing gardening frequency, even after adjustment for covariates. Median survival times were 88.5, 89.1, and 89.8 years (age– and sex–adjusted), and 88.4, 89.3, and 89.7 years (fully–adjusted, including physical activity) for never/rarely, sometimes, and frequent gardeners, respectively.

## 4. Discussion

This study provides long–term evidence that gardening in later life is associated with healthier aging and longevity. At age 79, higher gardening frequency was associated with better QoL and psychological wellbeing, stronger physical function (gait speed, lung function, grip strength), greater daily functioning, and longer telomeres. Across subsequent follow–ups, more frequent gardeners experienced slower declines in gait speed and slower telomere attrition between ages 79 and 90, and a 22 % lower risk of death over 25 years compared with those who never or rarely gardened. Although effect sizes were modest, associations remained robust after adjusting for sociodemographic, lifestyle, and physical activity factors. These findings provide some of the first long-term evidence linking this everyday activity to biological aging and survival outcomes in very old age.

Our results build on a growing body of evidence linking gardening with subjective wellbeing—a key aspect of mental health—in older adults (Gagliardi & Piccinini, 2019; Robson & Troutman-Jordan, 2015; Wang & MacMillan, 2013). Previous studies have reported positive cross–sectional associations between gardening and self–esteem, social connectedness, subjective happiness, and life satisfaction in older adults (Fjaestad et al., 2023; Machida, 2019; Scott et al., 2020) while qualitative and descriptive research indicates that gardening is an emotionally rewarding activity, offering purpose, structure, and fulfilment in daily life (Cheng et al., 2010; Scott et al., 2015). In our study, gardening was associated with higher overall and psychological QoL, along with fewer symptoms of anxiety and depression. These findings support prior evidence of psychosocial benefits associated with gardening in later life and highlight stress reduction and emotional restoration as potential mechanisms.

Regarding physical health, previous research suggests that older adults who garden may experience better balance, mobility, and a lower risk of falls (Chen & Janke, 2012), and small–scale interventions have shown improvements in mobility, hand dexterity, and reductions in anxiety and blood pressure, following guided gardening sessions (Hassan et al., 2018; Park et al., 2016b). Moreover, gardening has been demonstrated to provide sufficient physical activity intensity to meet recommended guidelines for older adults, supporting its role in promoting physical and mental health (Park et al., 2008). Systematic reviews, however, have found inconclusive evidence for physical health benefits, with mixed findings across observational and experimental designs (Nicklett et al., 2016; Wang & Boros, 2025). Our study contributes longitudinal evidence, using objective (directly measured) physical function, that gardening frequency is associated with slower decline in gait speed, a key marker of functional aging and predictor of adverse outcomes including frailty, hospitalisation, and mortality (Studenski et al., 2011). At baseline, gardening was also associated with better lung function, grip strength, and functional ability, indicating better day-to-day functional health. Given that even low intensity gardening requires mobility, strength, and dexterity, it is perhaps unsurprising that gardeners tend to report better physical function and fewer limitations in daily activities. Importantly, the observed associations with gait speed and functional measures remained after adjusting for overall physical activity, suggesting that gardening may confer specific benefits beyond those of general exercise, possibly through muscle engagement, balance training, and outdoor movement. The absence of longitudinal associations for some physical measures, such as grip strength or lung function, may reflect measurement limitations, lower sensitivity of these outcomes to gardening activity, or insufficient intensity or duration of exposure.

To our knowledge, this is the first study to examine gardening in relation to telomere dynamics. More frequent gardeners had longer telomeres at baseline and modestly slower telomere attrition over time, consistent with potential cellular-level benefits. These findings support the idea that gardening may influence not only subjective and functional aspects of aging but also fundamental biological processes. Mechanisms may include reduced inflammation and oxidative stress, as supported by previous research linking nature exposure, stress reduction, physical activity, and telomere maintenance (Espinosa-Otalora et al., 2021; Mohol et al., 2025; Puterman et al., 2018).

Frequent gardening was associated with a 22 % lower risk of death over 25 years of follow-up, contributing to the very limited existing evidence on gardening activity and mortality. One previous study reported reduced 10-year mortality among gardeners compared with non-gardeners using indirect risk estimates rather than observed deaths (Veldheer et al., 2023). In a larger study using registry-linked data from the NHANES cohort, Liang et al. (2024) found the same 22 % lower risk associated with higher gardening frequency among older adults, over a median 16.8-year follow-up, adjusting for individual-level confounders. The replication of this hazard ratio in our independent cohort—despite differences in population, follow-up duration, and adjustment for environmental quality—strengthens the evidence that gardening may have a meaningful protective effect on survival. Our study builds on this evidence by providing one of the longest verified mortality follow-ups to date, with adjustment for both individual- and area-level factors (i.e., neighbourhood quality). Such adjustment is important, as environmental conditions such as access to amenities can independently influence health outcomes and survival and may confound associations. Together, these findings suggest that gardening may support longevity, even in very old age, potentially via psychosocial, physical, and environmental pathways.

Nonetheless, we cannot exclude the possibility of residual confounding—healthier individuals with better functional capacity may be more inclined to garden and exhibit slower biological aging and live longer—highlighting the need for cautious interpretation. In undertaking the challenging task of using correlational data to give us indications about causality, we hypothesised that, if gardening frequency were simply a general biomarker of fitness, sociodemographic factors or lifestyle (which are also known to be associated with healthier aging outcomes), inclusion of those variables as covariates in our models would mostly explain the associations between gardening and our aging-related outcomes. Instead, we found limited attenuation in those associations, but we caution that there may well be other aspects of broad functional capacity that we have not measured or accounted for here.

Key strengths of the study include the longitudinal design, well-characterised and age-homogenous sample, and repeated measures of health over more than a decade. Objective indicators of physical function and biological aging, alongside verified mortality data in a sample with near-complete mortality ascertainment, improve the reliability of the data and the relevance of findings for long-term health. The availability of rich phenotypic data in LBC1921 allowed us to examine multiple domains of aging and adjust for a broad range of potential confounders, including individual- and area-level factors, increasing confidence in the observed associations, and allowing for a more nuanced understanding of how personal and environmental factors may shape the relationship between gardening and healthy aging.

Limitations include the observational design, which limits causal inference. Although the consistency of associations across multiple domains of health, and survival, coupled with longitudinal findings, supports the plausibility of a directional effect, reverse causation cannot be ruled out. Healthier individuals may be more likely to take up or maintain gardening, and this possibility should be considered when interpreting our and others’ results. Gardening frequency was self-reported at baseline only, with no data on type, intensity, or duration, limiting exploration of dose-response effects and inferences about long-term engagement. The sample was predominantly White Scottish and relatively healthy, which may limit generalisability to more diverse or socioeconomically disadvantaged populations.

While we lacked data on gardening type, most participants likely engaged in home gardening. However, some may have participated in community or allotment gardening, which can offer additional or distinct benefits beyond those typically associated with home-based activity. These include greater opportunities for social interaction, shared purpose, and physical exertion, particularly when travel to communal spaces and maintenance of shared plots are required. ‘Collective’ gardening also supports healthier eating through access to home-grown produce (Tharrey & Darmon, 2022), social cohesion and sense of community (Soga et al., 2017; Truong et al., 2022), and reduces loneliness (Van den Berg et al., 2011). Future research should consider gardening type and context to better understand how different forms of gardening may influence health and aging outcomes.

These findings have implications for aging research and public health. Gardening’s adaptability across different levels of health and mobility, and its capacity to support physical, social, and emotional engagement, make it a promising candidate for lifestyle-based interventions for older adults. Importantly, gardening emerged as a low-risk activity, with no evidence of adverse associations in the outcomes tested here. Because gardening is typically undertaken at home and can be adjusted to individual preference and ability, it aligns well with the principle of ‘aging in place’—the preference to maintain independence within one’s own community for as long as possible (Vanleerberghe et al., 2017). Public health strategies and age-friendly urban planning efforts that improve access to safe, well-designed outdoor spaces, and supportive infrastructure for gardening, may help extend these benefits to more diverse older populations.

### 4.1. Conclusion

In conclusion, this study provides evidence that gardening—a common, low-cost, and widely accessible behaviour—may contribute to healthier psychological, physical, and biological aging, and is associated with a meaningful reduction in mortality risk over decades. These results extend existing evidence by demonstrating that an everyday leisure activity is linked to long-term differences in functional and cellular aging in very old age. By identifying gardening as a feasible component of strategies that support independence and wellbeing, the findings highlight its potential relevance for public health, social prescribing, and environmental design.

In practice, these findings support the inclusion of gardening within health promotion and social prescribing initiatives for older adults, particularly those aiming to maintain mobility, wellbeing, and independence in later life. At a policy level, they underscore the importance of age-friendly housing and urban environments that enable access to safe, useable garden spaces. For research, the results highlight the need for more longitudinal, intervention, and quasi-experimental studies to test causality and determine the most beneficial forms of gardening and optimal exposure (frequency and duration) across diverse populations.

## Supplementary Material

1

## Figures and Tables

**Table 1:** Participant characteristics by gardening frequency.

Characteristic	Full sample	Never/rarely	Sometimes	Frequently	
	*n* = 475	*n* = 190	*n* = 78	*n* = 207	p
Continuous variables, mean (SD)					
Age	79.07 (0.6)	79.13 (0.6)	79.05 (0.5)	79.03 (0.6)	0.224
Years of education	10.91 (2.4)	10.40 (2.1)	11.29 (2.6)	11.22 (2.5)	<0.001
Body mass index	26.11 (4.2)	26.85 (4.5)	25.36 (4.0)	25.73 (3.8)	0.006
Physical activity	6.22 (8.5)	5.10 (8.1)	5.80 (8.2)	7.36 (8.8)	0.028
Neighbourhood quality	14.79 (3.0)	14.15 (2.8)	15.09 (3.0)	15.25 (3.1)	<*0.001*
Categorical variables, *n* (%)					
Sex					0.002
Female	271 (57.1 %)	127 (66.8 %)	40 (51.2 %)	104 (50.2 %)	
Male	204 (42.9 %)	63 (33.2 %)	38 (48.7 %)	103 (49.8 %)	
Social class					0.023
Class I (professional)	107 (22.6 %)	35 (18.6 %)	15 (19.2 %)	57 (27.5 %)	
Class 2	169 (35.7 %)	57 (30.3 %)	28 (35.9 %)	84 (40.6 %)	
Class 3	180 (38.1 %)	87 (46.3 %)	33 (42.3 %)	60 (29.0 %)	
Class 4	11 (2.3 %)	7 (3.7 %)	1 (1.3 %)	3 (1.4 %)	
Class 5 (manual)	6 (1.3 %)	2 (1.1 %)	1 (1.3 %)	3 (1.4 %)	
Lives alone, yes	226 (47.6 %)	105 (55.6 %)	30 (38.5 %)	91 (44.2 %)	0.015
Smoking status					0.985
Ever smoker	266 (56.0 %)	107 (56.3 %)	44 (56.4 %)	115 (55.6 %)	
Never smoker	209 (44.0 %)	83 (43.7 %)	34 (43.6 %)	92 (44.4 %)	
CVD, yes	141 (29.7 %)	58 (30.5 %)	26 (33.3 %)	57 (27.5 %)	0.601
Stroke, yes	41 (8.6 %)	19 (10.0 %)	2 (2.6 %)	20 (9.7 %)	0.112
Cancer, yes	45 (9.5 %)	14 (7.4 %)	7 (9.0 %)	24 (11.6 %)	0.352

*Note*: SD = standard deviation; CVD = cardiovascular disease.

**Table 2:** Longitudinal summaries of aging markers by age at wave of testing.

Aging marker	79 years (Wave 1) *n* = 475			83 years (Wave 2) *n* = 298			87 years (Wave 3) *n* = 198			90 years (Wave 4) *n* = 126		
	Mean	SD	*n*	Mean	SD	*n*	Mean	SD	*n*	Mean	SD	*n*
Quality of Life	63.92	7.3	472							61.48	6.5	122
Psychological Distress	8.58	4.6	475				8.26	4.7	198	7.65	4.1	126
Lung Function (FEV_1_)	1.90	0.6	472				1.75	0.5	198	1.64	0.5	125
Gait Speed (6m-walk)	4.67	1.7	470				6.46	3.3	184	6.72	2.7	93
Grip Strength	26.73	9.2	472	25.05	9.0	298	21.25	8.7	197	20.20	8.3	125
Functional Disability	2.16	2.7	475				4.05	4.0	198	5.14	4.6	125
Telomere Length	4.11	0.4	425				4.18	0.6	141	3.17	0.7	90
DNAm PhenoAge	76.07	7.9	378				77.00	7.7	170	79.92	7.6	80

*Note*: Quality of Life was measured at ages 79 and 90 only. Grip Strength was additionally measured at age 83.

**Table 3:** Associations of gardening frequency with aging markers: intercepts (baseline levels, age 79) and slopes (rates of change, age 79 to 90).

Outcome		Model 1 (age + sex)				Model 2 (multivariable)				Sensitivity Model (+ physical activity)			
	Parameter	Std β	SE	*p*	95 % CI	Std β	SE	*p*	95 % CI	Std β	SE	*p*	95 % CI
Quality of Life	Intercept	0.201	0.050	**<0.001**	[0.122, 0.318]	0.177	0.049	**<0.001**	[0.090, 0.283]	0.160	0.049	**<0.001**	[0.071, 0.263]
	Slope	0.048	0.104	0.604	[−0.152, 0.259]	0.058	0.105	0.545	[−0.144, 0.272]	0.054	0.105	0.568	[−0.147, 0.267]
Psychological Wellbeing^a^	Intercept	0.129	0.060	**0.032** †	[0.011, 0.247]	0.125	0.059	**0.034** †	[0.010, 0.240]	0.114	0.059	0.052	[−0.001, 0.229]
	Slope	−0.149	0.183	0.815	[−0.507, 0.209]	−0.101	0.153	0.510	[−0.401, 0.199]	−0.093	0.153	0.543	[−0.393, 0.207]
Lung Function	Intercept	0.114	0.034	**0.001**	[0.046, 0.181]	0.092	0.035	**0.009**	[0.023, 0.160]	0.081	0.035	**0.019**	[0.013, 0.149]
	Slope	0.047	0.083	0.572	[−0.116, 0.210]	0.072	0.102	0.483	[−0.128, 0.272]	0.075	0.101	0.455	[−0.122, 0.273]
Gait Speed^a^	Intercept	0.250	0.067	**<0.001**	[0.118, 0.381]	0.192	0.052	**<0.001**	[0.090, 0.294]	0.174	0.051	**0.001**	[0.074, 0.274]
	Slope	−0.206	0.087	**0.018**	[−0.376, −0.035]	−0.200	0.081	**0.013**	[−0.357, −0.081]	−0.197	0.081	**0.015**	[−0.354, −0.039]
Grip Strength	Intercept	0.074	0.030	**0.014**	[0.015, 0.133]	0.070	0.031	**0.022**	[0.015, 0.130]	0.062	0.030	**0.042** †	[0.002, 0.121]
	Slope	0.041	0.113	0.717	[−0.181, 0.263]	−0.015	0.108	0.893	[−0.227, 0.198]	−0.025	0.109	0.817	[−0.239, 0.189]
Functional Ability^a^	Intercept	0.332	0.091	**<0.001**	[0.155, 0.510]	0.293	0.078	**<0.001**	[0.140, 0.446]	0.267	0.073	**<0.001**	[0.125, 0.409]
	Slope	0.065	0.191	0.735	[−0.310, 0.440]	−0.060	0.161	0.708	[−0.256, 0.376]	0.037	0.150	0.804	[−0.256, 0.331]
Telomere Length^b^	Intercept	0.151	0.050	**<0.001**	[0.054, 0.249]	0.142	0.049	**0.004**	[0.045, 0.239]	0.148	0.049	**0.003**	[0.052, 0.245]
	Slope	−0.338	0.129	**0.009**	[−0.591, −0.084]	−0.252	0.090	**0.005**	[−0.429, −0.075]	−0.258	0.089	**0.004**	[−0.433, −0.083]
DNAm PhenoAge^a^	Intercept	0.090	0.067	0.181	[−0.042, 0.221]	0.070	0.067	0.299	[−0.062, 0.201]	0.068	0.067	0.314	[−0.064, 0.199]
	Slope	0.194	0.213	0.362	[−0.223, 0.600]	0.160	0.195	0.410	[−0.221, 0.542]	0.173	0.194	0.373	[−0.207, 0.503]

*Note*. Std β = standardised beta estimate; SE = standard error; CI = confidence intervals.

Estimates are derived from latent growth curve models for all outcomes except for Quality of Life (QoL). For QoL, estimates were derived from linear regression models, where intercept associations correspond to baseline (age 79) levels, and slope associations reflect change in QoL, derived by regressing age 90 scores on age 79 scores.

Outcomes were coded such that higher values indicate healthier aging.

Model 1: adjusted for age and sex; Model 2: additionally adjusted for education, social class, living alone, neighbourhood amenities, smoking, body mass index, cardiovascular disease, stroke, and cancer; Sensitivity: Model 2 plus physical activity.

*p*-values in bold-type are significant at the *p* <0.05 level;

† Does not survive False Discovery Rate (FDR) correction (α = 0.05).

a For reverse-coded measures (Psychological Wellbeing, Gait Speed, Functional Ability, DNAm PhenoAge), negative slopes indicate less decline or slower aging associated gardening over time.

b For Telomere Length, a negative slope reflects slower telomere attrition over time, corresponding to healthier aging.

**Table 4:** Cox proportional hazards models of 25-year mortality risk.

Ref group: Gardening: never/rarely	HR	SE	*p*	95 % CI
Gardening: sometimes (group 1)				
Model 1: age + sex	0.857	0.135	0.251	[0.657, 1.116]
Model 2: multivariable	0.833	0.142	0.200	[0.631, 1.101]
Sensitivity: + physical activity	0.796	0.143	0.111	[0.601, 1.054]
Gardening: frequently (group 2)				
Model 1: age + sex	0.784	0.103	**0.019**	[0.640, 0.961]
Model 2: multivariable	0.775	0.112	**0.023**	[0.622, 0.965]
Sensitivity: + physical activity	0.779	0.113	**0.027**	[0.624, 0.972]

*Note*: HR = hazard ratio; SE = standard error; CI = confidence intervals.

Two participants could not be traced for mortality linkage and were excluded (sample *n* = 473). At the end of the 25-year follow-up period, 469 participants were deceased and 4 were alive. Time-to-event was defined as the number of days from birth to death or censoring (March 2025).

Model 1: adjusted for age and sex; Model 2: additionally adjusted for education, social class, living alone, neighbourhood amenities, smoking, body mass index, cardiovascular disease, stroke, and cancer; Sensitivity: Model 2 plus physical activity.

*p*-values in bold-type are significant at the *p* < 0.05 level.

## Data Availability

Data from this study can be requested from the Lothian Birth Cohorts. Further information, including data request form, data summaries and data dictionaries can be found here: https://www.ed.ac.uk/lothian-birth-cohorts.

## Appendix A. Supplementary data

Supplementary data to this article can be found online at https://doi.org/10.1016/j.jenvp.2025.102889.
