# Supplementary material for: Gardening, healthy aging, and longevity: Longitudinal evidence from 25 years of the Lothian Birth Cohort 1921
Source: J Environ Psychol. Author manuscript; Available in PMC 2026 Jul 15. (PMC13367424; doi:10.1016/j.jenvp.2025.102889)
Supplement: 1 [file NIHMS2188337-supplement-1.docx]

**
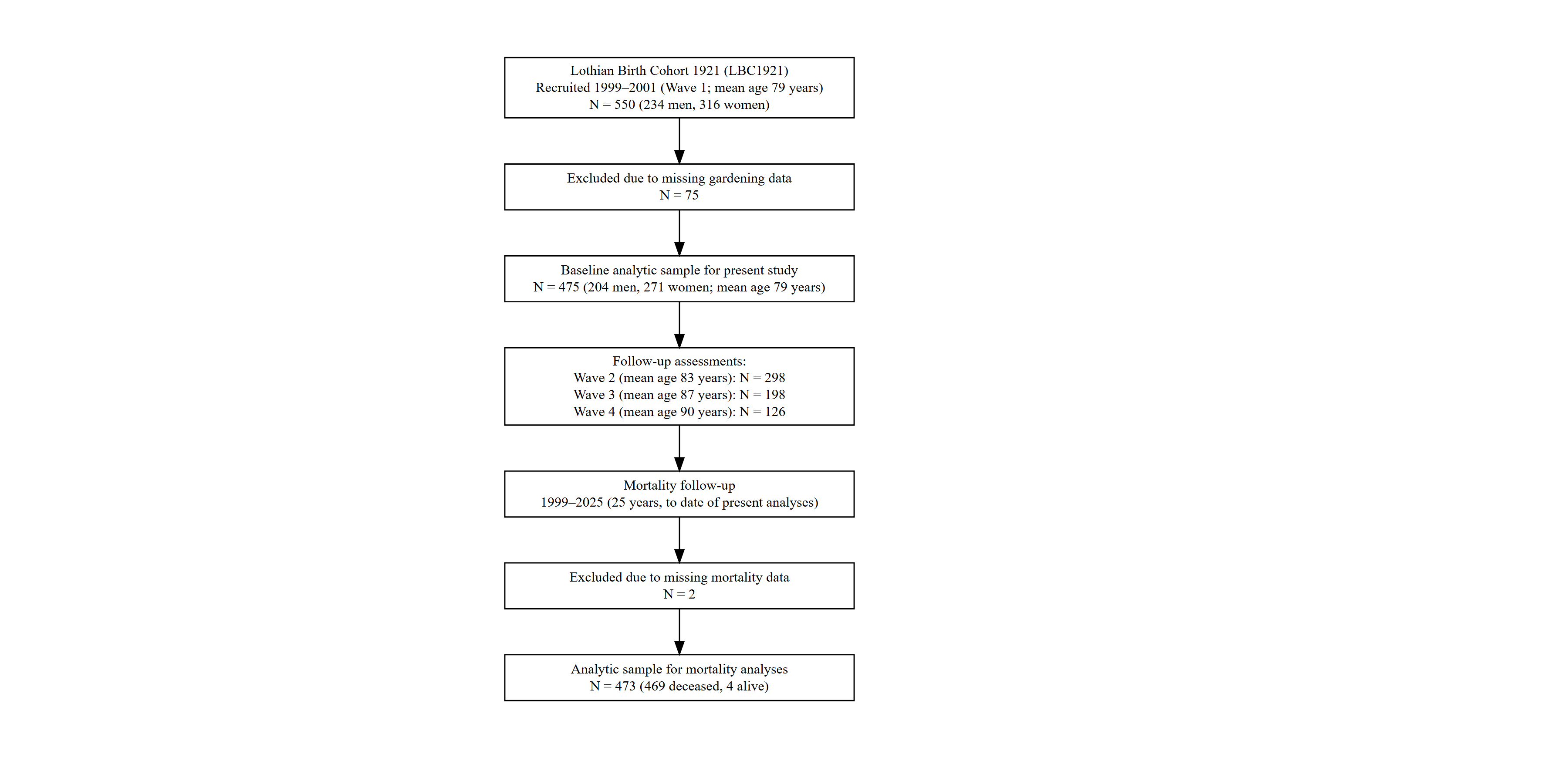
**

**Figure S1.** Flowchart of participant inclusion and follow-up in the Lothian Birth Cohort 1921

**Table S1.** Number of participants with missing data on covariate variables at baseline (age 79, wave 1, *n* = 475)

| Characteristic | Missing *n* |
| --- | --- |
| Age | 0 |
| Years of education | 2 (0.4%) |
| Body mass index | 3 (0.6%) |
| Physical activity (days/month) | 9 (1.9%) |
| Neighbourhood quality | 13 (2.7%) |
| Sex | 0 |
| Social class | 2 (0.4%) |
| Lives alone | 0 |
| Smoking status | 0 |
| Cardiovascular disease | 0 |
| Stroke | 0 |
| Cancer | 0 |

**Table S2**. Model fit indices for the final models of outcomes in Table 3

| Outcome | R^2^ (baseline) | R^2^ (change) | CFI | RMSEA | SRMR |
| --- | --- | --- | --- | --- | --- |
| Quality of Life^a^ | 0.146 | 0.145 |  |  |  |
| Psychological Wellbeing |  |  | 0.93 | 0.05 | 0.03 |
| Lung Function |  |  | 0.99 | 0.02 | 0.01 |
| Gait Speed |  |  | 0.92 | 0.04 | 0.07 |
| Grip Strength |  |  | 0.99 | 0.03 | 0.03 |
| Functional Ability |  |  | 0.97 | 0.06 | 0.04 |
| Telomere Length |  |  | 0.94 | 0.04 | 0.04 |
| DNAm PhenoAge |  |  | 0.90 | 0.06 | 0.03 |

Note: CFI = Comparative fit index; RMSEA = Root Mean Square Error of Approximation; SRMR = Standardised Root Mean Square Residual

^a^Quality of Life was measured at two time-points only so linear regression was used to test associations with gardening frequency (R^2^ model values are reported). For all other outcomes, model fit indices were derived from growth curve models which require a minimum of 3 waves of data.

**Table S3**. Associations of gardening frequency with Quality of Life sub-domains: intercepts (baseline levels, age 79) and slopes (rates of change, age 79 to 90)

|  |  | Model 1 (age + sex) | | | | Model 2 (multivariable) | | | | Sensitivity (+ physical activity) | | | |
| --- | --- | --- | --- | --- | --- | --- | --- | --- | --- | --- | --- | --- | --- |
| QoL sub-domain | Parameter | Std β | SE | P | 95% CI | Std β | SE | P | 95% CI | Std β | SE | P | 95% CI |
| Physical | Intercept | 0.204 | 0.050 | **<0.001** | [0.124, 0.321] | 0.186 | 0.050 | **<0.001** | [0.102, 0.300] | 0.167 | 0.050 | **<0.001** | [0.083, 0.278] |
|  | Slope | 0.025 | 0.101 | 0.782 | [-0.172, 0.228] | -0.001 | 0.102 | 0.996 | [-0.203, 0.201] | -0.003 | 0.102 | 0.974 | [-0.206, 0.199] |
| Psychological | Intercept | 0.140 | 0.051 | **0.003** | [0.054, 0.253] | 0.145 | 0.052 | **0.002** | [0.061, 0.263] | 0.140 | 0.052 | **0.004** | [0.047, 0.250] |
|  | Slope | 0.054 | 0.103 | 0.560 | [-0.143, 0.263] | 0.016 | 0.106 | 0.869 | [-0.192, 0.227] | 0.014 | 0.106 | 0.889 | [-0.195, 0.225] |
| Environmental | Intercept | 0.168 | 0.050 | **<0.001** | [0.085, 0.283] | 0.121 | 0.048 | **0.007** | [0.035, 0.225] | 0.108 | 0.048 | **0.016** | [0.021, 0.211] |
|  | Slope | 0.088 | 0.103 | 0.341 | [-0.105, 0.302] | 0.121 | 0.102 | 0.187 | [-0.067, 0.339] | 0.115 | 0.101 | 0.201 | [-0.071, 0.331] |
| Social | Intercept | 0.117 | 0.051 | **0.012** | [0.029, 0.228] | 0.086 | 0.051 | 0.079 | [-0.011, 0.191] | 0.074 | 0.051 | 0.131 | [-0.023, 0.179] |
|  | Slope | 0.089 | 0.102 | 0.328 | [-0.102, 0.304] | 0.099 | 0.105 | 0.311 | [-0.102, 0.316] | 0.096 | 0.105 | 0.323 | [-0.104, 0.314] |

*Note*. Std β = standardised beta estimate; SE = standard error; CI = confidence intervals; QoL = quality of life.
Estimates were derived from linear regression models, where intercept associations correspond to baseline (age 79) levels, and slope associations reflect change in quality of life, derived by regressing age 90 scores on age 79 scores and modeling the unstandardized residuals.

Higher values represent healthier aging.

Model 1: adjusted for age and sex; Model 2: additionally adjusted for education, social class, living alone, neighbourhood amenities, smoking, body mass index, cardiovascular disease, stroke, and cancer; Sensitivity: Model 2 plus physical activity.
*p*-values in bold-type are significant at the *p* < 0.05 level
